# Usability, Perceived Usefulness, and Shared Decision-Making Features of the AFib 2gether Mobile App: Protocol for a Single-Arm Intervention Study

**DOI:** 10.2196/21986

**Published:** 2021-02-24

**Authors:** Alok Kapoor, Andreza Andrade, Anna Hayes, Kathleen Mazor, Carl Possidente, Kim Nolen, Rozelle Hegeman-Dingle, David McManus

**Affiliations:** 1 Department of Medicine University of Massachusetts Medical School Worcester, MA United States; 2 Medical Affairs Pfizer Inc New York, NY United States

**Keywords:** shared decision making, mobile health, stroke risk, anticoagulation risk, anticoagulation education, atrial fibrillation, anticoagulation therapy, anticoagulation, atrial flutter, mobile phone

## Abstract

**Background:**

The Centers for Disease Control and Prevention has estimated that atrial fibrillation (AF) affects between 2.7 million and 6.1 million people in the United States. Those who have AF tend to have a much higher stroke risk than others. Although most individuals with AF benefit from anticoagulation (AC) therapy, a significant majority are hesitant to start it. To add, providers often struggle in helping patients negotiate the decision to start AC therapy. To assist in the communication between patients and providers regarding preferences and knowledge about AC therapy, different strategies are being used to try and solve this problem. In this research study, we will have patients and providers utilize the AFib 2gether app with hopes that it will create a platform for shared decision making regarding the prevention of stroke in patients with AF receiving AC therapy.

**Objective:**

The aim of our study is to measure several outcomes related to encounters between patients and their cardiology providers where AFib 2gether is used. These outcomes include usability and perceived usefulness of the app from the perspective of patients and providers. In addition, we will assess the extent and nature of shared decision making.

**Methods:**

Eligible patients and providers will evaluate the AFib 2gether mobile app for usability and perceived usefulness in facilitating shared decision making regarding understanding the patient’s risk of stroke and whether or not to start AC therapy. Both patients and providers will review the app and complete multiple questionnaires about the usability and perceived usefulness of the mobile app in a clinical setting. We will also audio-record a subset of encounters to assess for evidence of shared decision making.

**Results:**

Enrollment in the AFib 2gether shared decision-making study is still ongoing for both patients and providers. The first participant enrolled on November 22, 2019. Analysis and publishing of results are expected to be completed in spring 2021.

**Conclusions:**

The AFib 2gether app emerged from a desire to increase the ability of patients and providers to engage in shared decision making around understanding the risk of stroke and AC therapy. We anticipate that the AFib 2gether mobile app will facilitate patient discussion with their cardiologist and other providers. Additionally, we hope the study will help us identify barriers that providers face when placing patients on AC therapy. We aim to demonstrate the usability and perceived usefulness of the app with a future goal of testing the value of our approach in a larger sample of patients and providers at multiple medical centers across the country.

**Trial Registration:**

ClinicalTrials.gov NCT04118270; https://clinicaltrials.gov/ct2/show/NCT04118270

**International Registered Report Identifier (IRRID):**

DERR1-10.2196/21986

## Introduction

Atrial fibrillation (AF) and atrial flutter occur in epidemic proportions in the United States [1-4]. The Centers for Disease Control and Prevention estimates that AF affects between 2.7 million and 6.1 million people in the United States [5]. Anticoagulation (AC) is the mainstay of therapy, but many patients are reluctant to start taking anticoagulants [6]. Patients who have a diagnosis of AF generally have a higher risk of stroke than the general population [7]. Even among those who do start AC therapy, many do not persist with the treatment after bleeding or other setbacks. Providers also struggle with balancing the risks and benefits of AC therapy. Being able to determine an optimal decision for each patient is a valuable goal in stroke prevention. Shared decision making has been recommended by the American Heart Association (AHA) and other professional societies as a way to arrive at an optimal decision, but the usability and perceived usefulness of conducting shared decision making via an app visit are unclear [8].

Conducting shared decision-making visits will require several changes from standard provider-patient interactions. Shared decision-making visits will help patients and providers make the best choice in therapy that will fit into a patient’s life. Firstly, providers do not always draw attention to the fact that there is a decision to be made and may make the decision for the patient without soliciting the patient’s preferences. Secondly, providers do not always inquire about the patient’s preferred treatment approach, which can create a barrier for shared decision making. It is also possible that providers may not be knowledgeable or confident in managing AF patients with the most recently published guidelines and the advent of direct oral AC therapy (ie, modern AF management). Conducting AF management through a shared decision-making process may help in overcoming the above limitations. Currently, it is unknown how best to operationalize shared decision making around AC therapy for AF.

Shared decision-making tools can help patients make informed decisions with less conflict [9]. The AFib 2gether mobile app, which was developed by Pfizer Inc in consultation with a cardiologist (DM), is one potential approach for operationalizing shared decision making around AC therapy for AF. The app can provide a platform for a patient to determine their risk of stroke and identify items for discussion at an upcoming visit with their provider. The app was designed to support collaboration during patient visits, which allows for the app to provide a high-level overview of AF and AC therapy and to prepare patients to have the tools needed to ask questions of their providers. The provider can review the answers given by the patient’s app as well as any questions the patient solicited in the app for discussion prior to the visit. The goal is that this interaction will help the patient make a more informed decision by helping them become more engaged in their health status and improve their stroke prevention management related to AF. We have not previously tested the app with patients and providers for usability and perceived usefulness for clinical encounters. Therefore, we describe herein our protocol for testing the app in a clinical setting, including measurement of the usability of the app and its usefulness during clinical appointments between patients with AF, who are not currently prescribed AC therapy, and their cardiology providers. We also propose a measurement of the extent and nature of shared decision making that occurs through audio-recording the encounters facilitated by our app.

## Methods

### Study Aims

The aim of the AFib 2gether research study is to measure the usability and perceived usefulness of the shared decision-making mobile app AFib 2gether from the perspective of patients and providers. We will also measure patient AC status by chart review 6 months after their shared decision-making visit to see if patients started AC therapy. Finally, we will assess the extent and nature of shared decision making through a review of audio-recordings of the patient encounters with their providers.

### Study Population

#### Setting

This study will take place at the cardiology practice of an academic, tertiary care health system in central Massachusetts.

#### Providers

We will enroll up to 20 cardiology providers practicing at the University of Massachusetts (UMass) Memorial Healthcare System in the Ambulatory Care Center (ACC) Cardiology Clinic.

#### Patients

We will enroll up to 60 patients who are not receiving AC therapy, with each provider contributing up to 6 AF patients with elevated stroke risk. Recruitment will be restricted to patients aged 18 years and older. To identify patients, we will use a diagnostic concept grouper within our electronic health record (EHR) system that follows our inclusion criteria consistent with AF. Subsequently, patients will be filtered to retain those patients with CHA_2_DS_2_-VASc stroke risk scores of 2 or greater [8] who were not on AC therapy and had an upcoming cardiology visit in the next 3 months. The CHA_2_DS_2_-VASc score assigns 1 point for congestive heart failure, hypertension, age 65 to 74 years, diabetes mellitus, vascular disease history, and female sex. The score assigns 2 points for age greater than 75 years and for previous stroke or transient ischemic attack history.

**Figure 1:**
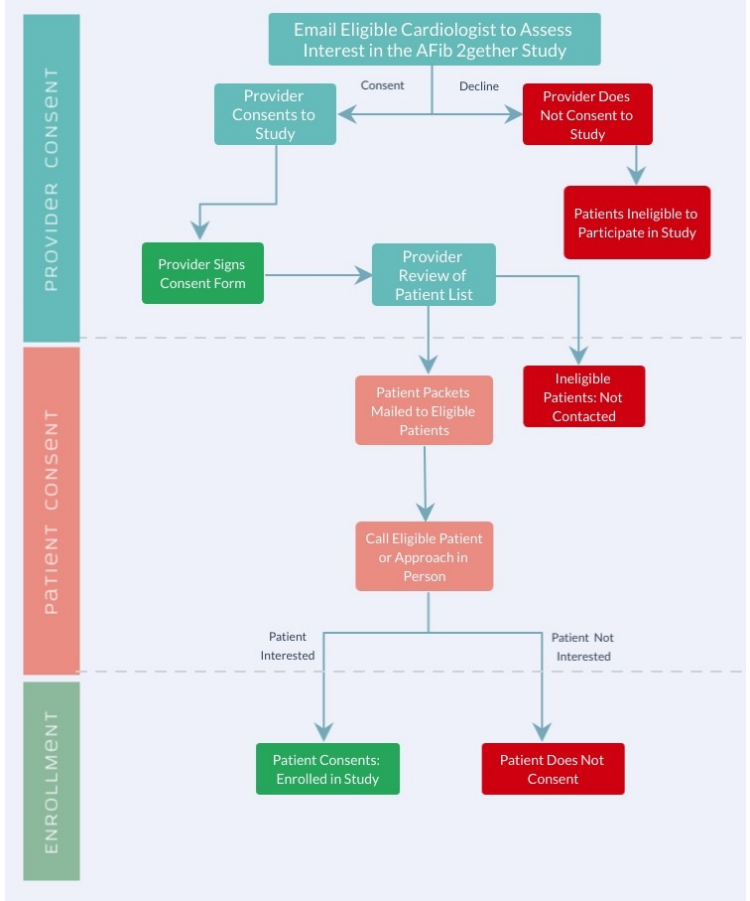
Overview of the provider and patient eligibility process for consent and enrollment in the study.

The following participants will be excluded: patients that have a WATCHMAN device or have had left atrial appendage closure surgery, patients in hospice or for whom life expectancy is less than 6 months, and patients with bleeding episodes or falls with injury 4 weeks prior to their cardiology appointment. Additionally, patients with preferred languages other than English will be excluded from the study because the app AFib 2gether is only available in English. Patients will be excluded from the study if they are members of vulnerable populations (ie, pregnant women and prisoners). The eligibility process is outlined (see Figure 1) to represent how patient eligibility will be verified before patients consent to participate in the study.

### Study Procedures

#### Screening and Recruitment

A custom query developed by the information technology department at our institution will be used to identify eligible patients who have encounters with consenting cardiology providers in the upcoming 3 months. A manual review of patients’ charts will be conducted to confirm that a patient was not receiving AC therapy (eg, from an outside provider, sometimes documented in scanned notes as opposed to structured variables).

The research assistant (RA) will start recruiting providers who had 3 or more patients that fit the study inclusion criteria. Each provider will then receive a study inquiry email from the RA to see if they are interested in participating in the study. If the provider agrees, they will sign the consent form and a letter will be mailed to their patients. Once enrolled, providers will receive a link to the secure REDCap (Research Electronic Data Capture)-based survey [10] to self-administer a questionnaire (see Multimedia Appendix 1) about their knowledge regarding AF management.

Additionally, letters signed by the patient’s cardiology provider along with a fact sheet and Health Insurance Portability and Accountability Act (HIPAA) authorization form will be mailed to each eligible patient 1 to 2 weeks prior to the patient’s appointment. Approximately 5 days later, the RA will call patients for a follow-up to gauge interest in participating in the study. For patients who scheduled a visit within 1 week of the appointment date, messages with study recruitment and consent materials will be sent to the patients as attachments using the Epic patient portal. Subsequently, patients will be called in the next 1 to 2 days to obtain consent. For patients who did not respond to either of these mechanisms or for patients that were scheduled within 24 hours of their appointment, our Institutional Research Board approved the RA to meet and recruit patients, as feasible, in the waiting room of the cardiology clinic prior to the patient visits.

#### Intervention and App Description

**Figure 2:**
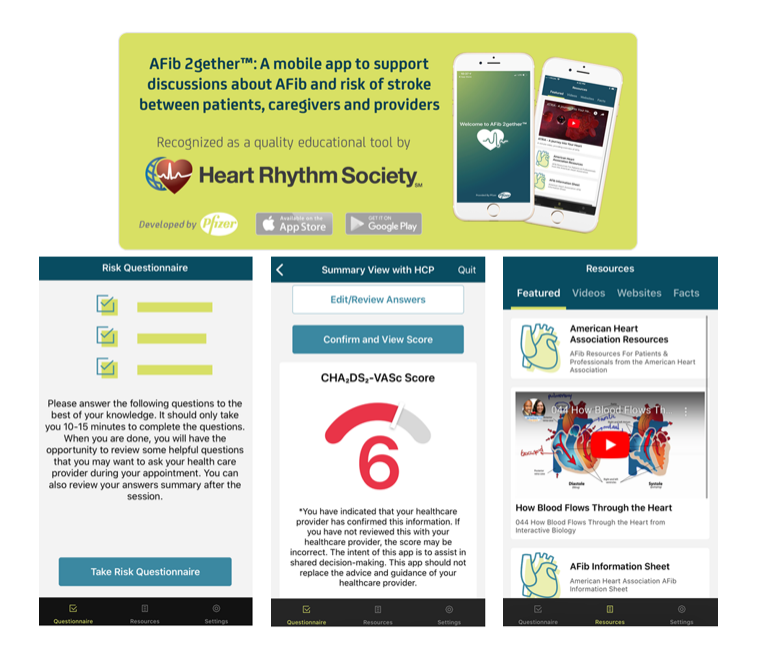
Advertisement of the AFib 2gether app in the Heart Rhythm Society’s patient toolkit with screenshots of sections of the app.

Once a patient has provided informed consent to participate, we will ask them to download the AFib 2gether mobile app onto their personal smartphone or a family member’s smartphone or to use the study device for in-person visits. AFib 2gether is a mobile app that may be helpful to foster a shared decision-making discussion between patients and providers. The AFib 2gether app may help increase a patient’s understanding of their risk of stroke due to AF through the personalized stroke risk calculator, information sheets, videos, website links, and facts in the app (see Figure 2). In addition, the app allows patients to select questions about AF and their stroke risk score to discuss with their health care providers. The goal of the app is to improve patient understanding; it stands in distinction to other apps related to assisting providers with choosing a particular anticoagulant.

For patients recruited at the time of their office visit, the RA will offer the study smartphone, an Android OS smartphone, for patient use with the AFib 2gether app predownloaded on the device or will assist the patient in downloading the app onto their personal device. Next, the RA will instruct the patient to put in a study-specific code for the app. Once the patient puts in the study code, the patient will be able to consent to the app’s terms and conditions. Once the patient has agreed to the app’s terms and conditions, the patient may then answer questions on the app to determine their knowledge of their stroke risk score. The questions include whether the patient has ever had heart failure, hypertension, diabetes, stroke, vascular disease, or previous stroke and what their age and gender are. Once the participant answers the questions, the app will display their categories for stroke risk and will allow the participant to select up to three questions from a list of 13 commonly asked questions that they may want to discuss with their provider during their visit based on their risk assessment. Examples of the types of questions a participant could choose are as follows: Would you like to know more about your condition? What is the cost of AC therapy? and What are the benefits of going on AC therapy to avoid stroke? The participant will be given the option to type in any additional questions they may have that were not listed in the app. Lastly, the patient’s risk factors and questions will then be sent via email to the provider as a PDF document to review prior to their appointment.

#### Data Collection

The RA will administer a modified version of the Mobile App Rating Scale (MARS) for the purpose of assessing usability (see Multimedia Appendix 2) [11]. The MARS is a validated survey that can be used to assess health apps [11]. The modified questionnaire includes functionality and aesthetics domains that are similar to the original. We will not include items from the engagement and entertainment or information domains. We will include one item from the app subjectivity quality domain, which is the overall star rating of the app. This followed recent evidence suggesting the validity of scoring each domain separately [12]. The RA will wait with the participant until their visit with the provider begins. Once the cardiology provider is ready to begin the visit, the RA will then turn on and place the encrypted recorder—the Olympus DS-7000 (OM Digital Solutions)—in the room. At this point, the RA will prompt the provider to review the questions posed by the patient and then step outside the room and allow the visit to take place without any further scripting.

At the end of the visit, the RA will collect the voice recorder and administer questionnaires to both the patient and provider related to the perceived usefulness of the app following the technology acceptance model (TAM) [13,14]. Although we did also consider the unified theory of acceptance and use of technology 2 (UTAUT2), which unifies the eight theories including the TAM, we selected the TAM as our theoretical construct given its applicability to individual patients following other examples in the literature. Accordingly, we will administer a questionnaire assessing the patient perceived usefulness of the mobile app (see Multimedia Appendix 3). For providers, we will administer a questionnaire assessing the provider perceived usefulness of the mobile app (see Multimedia Appendix 4).

Once the patient completes their appointment and questionnaires, the RA will give the patient a US $25 Amazon gift card for participating in the study. Providers will be compensated with a US $200 Amazon gift card after they complete all study activities.

The UMass Medical School approved our protocol, including data collection and incentive procedures.

#### Modified Study Procedures for COVID-19

During periods when in-person recruitment is not permissible due to COVID-19 restrictions, we will utilize remote recruitment and consenting processes. For remote patient recruitment, the same provider letter will be used, but the fact sheet and HIPAA authorization will be modified to remove information about the audio-recording. We will not replace audio-recording with another method of objectively measuring the extent and type of shared decision making that occurs. The original HIPAA authorization form’s wet signature will be replaced with a process of acknowledgment to disclose protected health information through the process of agreeing to participate in the study and phone interview.

### Primary Outcomes

#### Usability

We will group items in the MARS into three domains for functionality, aesthetics, and overall quality; they will receive a rating of number of stars out of 5.

#### Perceived Usefulness

Perceived usefulness will be calculated for patients and providers based on a custom set of questions derived from the TAM. More specifically, we will examine their distribution similar to how we described the usability outcome. We collected usefulness data on a 5-point Likert scale for simplicity’s sake, in contrast to the traditional 7-point Likert scale used in TAM or UTAUT2 literature. In addition, timing will be recorded for each of the components of the shared decision-making encounter. The distribution of time required for each activity will be reviewed.

#### Shared Decision Making

We will also assess for elements of shared decision making, including multiple themes covered in established instruments [15]. These will include a mention that options are available, evidence that the provider shared stroke and bleeding risk with the patient, and, most notably, evidence of patient involvement in the discussion.

### Secondary Outcome: Anticoagulation Start

The RA will review each patient’s medical record in our institution’s EHR system, Epic, to see if the patient started AC therapy within these 6 months.

### Patient and Provider Characteristics

#### Patient Demographics

Through electronic capture from the data repository associated with our institution’s EHR system, we will collect age, sex, race, and ethnicity information.

#### Comorbidities

From our EHR system, we will also collect the CHA_2_DS_2_-VASc scores using our previously validated algorithm. From manual chart review, we will also collect information about why the patient did not previously receive AC therapy, including potential responses, such as *low AF burden*, *the patient refused*, *fall risk*, and *concomitant aspirin use*.

#### Provider Factors

Through information available from our credentialing office, we will collect provider age and years in practice in addition to provider credentials (ie, MD versus NP or PA).

#### Provider Knowledge

We will measure provider AC therapy decision-making confidence in several areas, including applying guidelines from the ACC, the AHA, and the Heart Rhythm Society and assessing antithrombotic therapy, using CHA_2_DS_2_-VASc scores to access stroke risk, among others. Response options will include *somewhat confident*, *moderately confident*, and *very confident*.

### Analysis

For each domain of usability of the app (ie, MARS items), we will calculate the mean and standard deviation. For perceived usefulness, we will group patients into consolidated ordinal categories based on the Likert response format. We will then assess for trends in associations and examine associations between patient characteristics and usability and perceived usefulness. Where feasible, given low sample size, we will also calculate *t* tests or chi-square tests for determining statistical significance. We will perform all analyses in SAS 9.4 (SAS Institute Inc) [16]. The UMass Medical School Institutional Review Board approved our protocol.

## Results

This study was registered at ClinicalTrials.gov (NCT04118270). Enrollment in the AFib 2gether shared decision-making study is still ongoing for both patients and providers. The first participant enrolled on November 22, 2019. Analysis and publishing of results are expected to be completed in spring 2021.

## Discussion

We have developed a protocol to measure the usability and perceived usefulness of a mobile app to facilitate shared decision making for patients with AF not currently receiving AC therapy. We also describe the administration of a separate provider survey that will allow us to measure the association between provider knowledge and each of these outcomes. Our protocol provides flexibility to recruit patients during the COVID-19 pandemic or other circumstances where face-to-face interaction is not possible and where telehealth virtual engagement strategies are implemented.

With the fast-growing use of online sources for accessing health information throughout society, a shared decision-making app or tool for clinical settings has a great potential for impact. Man-Song-Hing et al developed a decision aid based on a risk stratification scheme that helped patients and providers make informed decisions about whether to use warfarin compared with aspirin for patients with AF [17]. More patients in the intervention group (n=138, 99%) were able to make definite choices regarding antithrombotic therapy compared with those in the control group (n=139, 94%; *P*=.02). More recently, Kunneman et al tested a shared decision-making tool that provided individualized risk estimates of stroke in various AC therapy options [18]. Although they did not find a significant effect on treatment decisions, more clinicians were satisfied with the encounter in the intervention arm compared with the standard arm. Neither of the two studies specifically studied the usability or perceived usefulness of their shared decision-making tool [17,18].

Overall, there are advantages and disadvantages to using mobile health apps to conduct shared decision making. Some of the potential advantages of using a mobile app for shared decision making include patient empowerment, encouragement of patient participation in medical decision making, and increased overall satisfaction [19]. However, we need to balance this against the potential to increase the anxiety of patients, security concerns, and lack of accessibility in lower-income areas. The AFib 2gether app provides a convenient and comfortable way for patients to identify concerns they have about initiating or resuming AC therapy. The AFib 2gether app provides an updated and accurate shared decision-making tool that is readily available to patients and providers through both Google Play and the Apple App Store, for Android phones and iPhones, respectively. The AFib 2gether app will provide the tools to patients and providers to help them make informed decisions about the best treatment options for the patients. Users of the app can refer to multiple reliable educational videos, websites, and facts about AF, stroke, and AC therapy.

We acknowledge multiple limitations to our work. Firstly, the sample size of both the provider and patient populations are too small to make any firm conclusions about clinical outcomes, such as initiating AC therapy. Therefore, we restricted the scope of the proposed study to verify the usability and perceived usefulness of the AFib 2gether mobile app, as well as the extent and nature of shared decision making with the use of the app. Secondly, we did not have a control population against which to compare our intervention. In the future, we plan to increase the sample size and conduct a randomized controlled trial powered to find a difference in AC therapy starts. Thirdly, some patients downloaded and explored the app at home prior to the visit, whereas others, such as those without smartphones, only reviewed it in the waiting room of the office. We invited the latter patients to arrive 30 minutes prior to their visit in order to adequately evaluate the app. Nonetheless, we acknowledge the variability in usability that might be reported for a patient who reviewed the app without time constraints at home compared to a patient who only reviewed the app in the waiting room. In the future, with a greater sample size, we plan to measure the discrete effect of the intervention in each situation. Fourthly, we also acknowledge that prompting by the RA may have made the app appear more useful than it would have appeared with no prompting. Given that we are still making minor modifications to enhance the app, we anticipate being able to replace the manual prompting with an automated text message alert reminding providers to review patient responses. Lastly, a final limitation is that the patients who agree to participate likely represent a healthier, more technologically proficient, and potentially more educated population. We did not collect specific information to gauge patient technology proficiency, so we cannot compare our population with others. Future assessment of technology proficiency would help us to understand the representativeness of the population exposed to the app versus the general AF population.

In conclusion, we have described the protocol for assessing the usability and perceived usefulness of a mobile app to facilitate shared decision making concerning AC therapy in patients with AF and elevated stroke risk. The AFib 2gether app-based intervention improves on other shared decision-making interventions by leveraging a convenient platform (ie, the cell phone app) and soliciting items for discussion and review before the patient-provider visit. Although we will not be able to confirm the ability of the app to demonstrate a significant increase in AC therapy starts, our study will lay the groundwork for future efforts to conduct a multicenter, randomized clinical trial that will be able to elucidate the impact of our mobile app on clinical outcomes. With the latter road paved, we anticipate generating significant interest among other researchers developing app-based interventions to facilitate shared decision making for AC therapy in AF and similarly challenging treatment decisions.

## Appendix

Multimedia Appendix 1 Assessment of provider knowledge and therapeutic approaches for reducing stroke risk in patients with nonvalvular atrial fibrillation.

Multimedia Appendix 2 Provider and patient usability of the mobile app.

Multimedia Appendix 3 Questionnaire assessing the patient perceived usefulness of the mobile app.

Multimedia Appendix 4 Questionnaire assessing the provider perceived usefulness of the mobile app.

## Abbreviations

AC: anticoagulation
ACC: Ambulatory Care Center
AF: atrial fibrillation
AHA: American Heart Association
CHA_2_DS_2_-VASc: congestive heart failure, hypertension, age ≥75 years, diabetes mellitus, prior stroke, vascular disease, age 65-74 years, sex category
EHR: electronic health record
HIPAA: Health Insurance Portability and Accountability Act
MARS: Mobile App Rating Scale
RA: research assistant
REDCap: Research Electronic Data Capture
TAM: technology acceptance model
UMass: University of Massachusetts
UTAUT2: unified theory of acceptance and use of technology 2
