# Supplementary material for: Usability, Perceived Usefulness, and Shared Decision-Making Features of the AFib 2gether Mobile App: Protocol for a Single-Arm Intervention Study
Source: JMIR Res Protoc. 2021 Feb 24;10(2):e21986. doi: 10.2196/21986 (PMC7946587; doi:10.2196/21986)
Supplement: Multimedia Appendix 1 [file resprot_v10i2e21986_app1.pdf]

17762

Assessment of Provider Knowledge and Therapeutic Approaches for Reducing Stroke Risk in Patients with Nonvalvular Atrial Fibrillation.

Any responses provided will be kept CONFIDENTIAL. Your participation is voluntary. All responses will be combined, and the information you provide will remain ANONYMOUS. Our facility may use a 3rd party to collate and report data from your responses. Completion of this form provides permission to do this. Please completely shade in each response oval that you select. Shade ovals like this ⬬. In the boxes, print neatly and write only 1 digit per box, for example: [2][3] . Thank you for your participation!

Healthcare Professional Type: ○ Physician ○ PA ○ NP ○ Nurse ○ Pharmacist ○ Other-Describe [ ]

Practice Area: ○ Cardiology ○ Hematology ○ Family Medicine ○ Neurology ○ Internal Medicine ○ Geriatrics ○ General Practice ○ Clinical Cardiac Electrophysiology ○ Interventional Cardiology ○ Other: [ ]

Practice Setting: (Check all that apply)

○ Group Practice ○ Hospital/IDS ○ Outpatient Clinic ○ Inpatient Clinic ○ Long-Term Care ○ Anticoagulation Clinic ○ Medical School ○ Other: [ ]

How long have you been in practice (in years): [ ][ ]

Approximately what percentage of your adult patients has a diagnosis of NVAF? (Mark one only):

○ None ○ 1-10 ○ 11-25 ○ 26-50 ○ 51+ ○ Don't know

Confidence in Managing Patients with NVAF

In managing patients with NVAF:	Not confident	Somewhat confident	Moderately confident	Very confident
1. How confident are you in using information about stroke risk and bleeding risk assessment to determine appropriate antithrombotic therapy?	○	○	○	○
2. How confident are you in selecting appropriate anticoagulant therapy?	○	○	○	○
3. How confident are you in using oral anticoagulant therapies for reducing the risk of stroke?	○	○	○	○
4. How confident or familiar are you in using CHA_2DS_2-VASc scores to access stroke risk?	○	○	○	○
5. How confident are you in applying ACC/AHA/HRS guidelines to the management of NVAF?	○	○	○	○
6.How confident or familiar are you in using HAS-BLED score to assess bleeding risk?	○	○	○	○

Form ID: [ ][ ][ ][ ] Site ID: [ ][ ][ ][ ]

Knowledge of NVAF Management

7a. What CHA_2DS_2 -$VASc_2$ score would you give for the following patient case?

JD is a 73yo male with HTN and CHF. His BP is 165/90. He has no history of a stroke or thromboembolic event.

- ○ CHA_2 DS_2VASc=1
- ○ CHA_2 DS_2VASc=2
- ○ CHA_2 DS_2VASc=3
- ○ CHA_2 DS_2VASc=4
- ○ CHA_2 DS_2VASc=5
- ○ I don't know the CHA_2DS_2-VASc score

7b. Which antithrombotic would you choose for this patient (choose all that apply)?

- ○ No therapy
- ○ Aspirin 81mg
- ○ Vitamin K Antagonist (VKA)
- ○ Direct Oral Anticoagulant (DOAC)

8. According to the current ACC/AHA/HRS guidelines, in which of the following NVAF patients over 65 years of age is aspirin alone an acceptable option for anticoagulation? (choose all that apply)

- ○ Individuals with diabetes
- ○ Female gender
- ○ Individuals with previous stroke or TIA
- ○ Individuals with heart failure
- ○ None of the above
- ○ Unsure

9. Please rate your knowledge with the following anticoagulant therapeutic classes in terms of their potential use in NVAF patients at risk for stroke:

	Not at all Familiar	Somewhat Familiar	Very Familiar
1. Vitamin K Antagonists (VKA)?	○	○	○
2. Direct Oral Anticoagulant (DOAC)?	○	○	○
