# Supplementary material for: Usability, Perceived Usefulness, and Shared Decision-Making Features of the AFib 2gether Mobile App: Protocol for a Single-Arm Intervention Study
Source: JMIR Res Protoc. 2021 Feb 24;10(2):e21986. doi: 10.2196/21986 (PMC7946587; doi:10.2196/21986)
Supplement: Multimedia Appendix 2 [file resprot_v10i2e21986_app2.docx]

**Provider and Patient Usability of Mobile App**

(To be administered to each patient after each patient encounter and to each provider after their first and last encounters)

1. **Performance: How accurately/fast do the app features (functions) and components**

**(buttons/menus) work?**

- 1. App is broken; no/insufficient/inaccurate response (e.g. crashes/bugs/broken features, etc.)
  2. Some functions work, but lagging or contains major technical problems
  3. App works overall. Some technical problems need fixing/Slow at times
  4. Mostly functional with minor/negligible problems
  5. Perfect/timely response; no technical bugs found

1. **Ease of use: How easy is it to learn how to use the app; how clear are the menu labels/icons and instructions?**
   1. No/limited instructions; menu labels/icons are confusing; complicated
   2. Usable after a lot of time/effort
   3. Usable after some time/effort
   4. Easy to learn how to use the app (or has clear instructions)
   5. Able to use app immediately; intuitive; simple

1. **Navigation: Is moving between screens logical/accurate/appropriate/ uninterrupted; are all necessary screen links present?**
   1. Different sections within the app seem logically disconnected and random/confusing/navigation

is difficult

- 1. Usable after a lot of time/effort
  2. Usable after some time/effort
  3. Easy to use or missing a negligible link
  4. Perfectly logical, easy, clear and intuitive screen flow throughout, or offers shortcuts

1. **Are interactions (taps/swipes/scrolls) consistent and intuitive across all components/screens?**
   1. Completely inconsistent/confusing
   2. Often inconsistent/confusing
   3. OK with some inconsistencies/confusing elements
   4. Mostly consistent/intuitive with negligible problems
   5. Perfectly consistent and intuitive

1. **Layout: Is arrangement and size of buttons/icons/menus/content on the screen appropriate or zoomable if needed?**
   1. Very bad design, cluttered, some options impossible to select/locate/see/read device, display not optimized
   2. Bad design, random, unclear, some options difficult to select/locate/see/read
   3. Satisfactory, few problems with selecting/locating/seeing/reading items or with minor screen size problems
   4. Mostly clear, able to select/locate/see/read items
   5. Professional, simple, clear, orderly, logically organized, device display optimized. Every design component has a purpose

1. **Graphics: How high is the quality/resolution of graphics used for buttons/icons/menus/content?**
   1. Graphics appear amateur, very poor visual design ‐ disproportionate, completely stylistically inconsistent 2 Low quality/low resolution graphics; low quality visual design – disproportionate, stylistically inconsistent 3 Moderate quality graphics and visual design (generally consistent in style)
   2. High quality/resolution graphics and visual design – mostly proportionate, stylistically consistent
   3. Very high quality/resolution graphics and visual design ‐ proportionate, stylistically consistent throughout

1. **Visual appeal: How good does the app look?**
   1. No visual appeal, unpleasant to look at, poorly designed, clashing/mismatched colors
   2. Little visual appeal – poorly designed, bad use of color, visually boring
   3. Some visual appeal – average, neither pleasant, nor unpleasant
   4. High level of visual appeal – seamless graphics – consistent and professionally designed
   5. As above, plus very attractive, memorable, stands out; use of color enhances app features/menus
2. **What is your overall star rating of the usability of the app?**
   1.  One of the worst apps I’ve used
   2.  
   3.    Average
   4.    
   5.      One of the best apps I’ve used
