# Supplementary material for: Usability, Perceived Usefulness, and Shared Decision-Making Features of the AFib 2gether Mobile App: Protocol for a Single-Arm Intervention Study
Source: JMIR Res Protoc. 2021 Feb 24;10(2):e21986. doi: 10.2196/21986 (PMC7946587; doi:10.2196/21986)
Supplement: Multimedia Appendix 4 [file resprot_v10i2e21986_app4.docx]

(To be administered after each patient encounter)

Please report your level of agreement with the proposed statements

**1. The app improved my understanding of patient’s preferences regarding anticoagulation?**

a. Strongly disagree

b. Somewhat disagree

c. Neutral

d. Somewhat agree

e. Strongly agree

**2. The app will save me time in focusing on those items which are most important to patients?**

a. Strongly disagree

b. Somewhat disagree

c. Neutral

d. Somewhat agree

e. Strongly agree

**3. The app will help me decide if my patient needs to be on anticoagulation.**

a. Strongly disagree

b. Somewhat disagree

c. Neutral

d. Somewhat agree

e. Strongly agree
